# Sleep recovery ameliorates submandibular salivary gland inflammation associated with paradoxical sleep deprivation in male Wistar rats

**DOI:** 10.1590/1678-7757-2024-0133

**Published:** 2024-01-13

**Authors:** Jude Ijuo Abeje, Shehu-Tijani T. Shittu, Olayinka Olawale Asafa, Bimpe Bolarinwa, Taye J. Lasisi

**Affiliations:** 1 University of Ibadan College of Medicine Department of Physiology Ibadan Nigeria University of Ibadan, College of Medicine, Department of Physiology, Ibadan, Nigeria.; 2 University of Ibadan College of Medicine Department of Oral Pathology Ibadan Nigeria University of Ibadan, College of Medicine, Department of Oral Pathology, Ibadan, Nigeria.

**Keywords:** Sleep deprivation, Saliva, Inflammation, Cytokines, Submandibular gland

## Abstract

Submandibular salivary gland inflammation has been suggested as one of the mechanisms underlying impaired salivary secretion associated with sleep deprivation (SD). However, whether the salivary inflammatory response occurs to the same extent in paradoxical sleep deprivation with or without sleep recovery remains unknown.

**Objective:**

This study evaluated the extent to which inflammation influences salivary impairments associated with paradoxical sleep deprivation with or without sleep recovery.

**Methodology:**

Male Wistar rats were randomly assigned into three groups as control, partial SD (PSD) with sleep recovery for four hours a day and total SD (TSD). Paradoxical SD was carried out for seven days in the SD groups, after which saliva, blood, and submandibular gland samples were taken. Levels of interleukin-6 (IL-6), tumour necrosis factor-alpha (TNF-α), and nitrite were determined in saliva, serum, and the submandibular salivary gland. Leucocyte count and neutrophil-lymphocyte ratio were determined in all groups. One-way ANOVA and the Tukey's post hoc tests were used for data analysis. P-values < 0.05 were considered statistically significant.

**Results:**

Levels of TNF-α, IL-6, and nitrite in the submandibular salivary glands were significantly higher in the TSD groups (p=0.04,p<0.001, p=0.03, respectively) than in the control. Saliva level of TNF-α was higher in the PSD and TSD groups (p=0.003 and p=0.01 respectively) than in the control. Neutrophil-lymphocyte ratio was significantly higher in both PSD and TSD groups than in the control (p<0.01 for both).

**Conclusion:**

While total SD produced higher inflammatory response in the submandibular salivary gland, four-hour sleep recovery ameliorated this impact. This finding suggests that sleep recovery is crucial to improve inflammatory salivary gland dysfunction induced by sleep deprivation.

## Introduction

Saliva is mainly secreted by the paired major salivary glands, namely the parotid, submandibular, and sublingual salivary glands, with contributions from a number of minor salivary glands in the oral cavity and the upper respiratory tract. The saliva these glands produce serves a variety of functions, including dental hygiene, antimicrobial activities, remineralization of dental hard tissues, repair of soft tissue injuries, and digestion. Previous studies have linked several factors and conditions affecting salivary secretion to different pathologies involving the salivary glands.^1,2^ For instance, decreased salivary secretion associated with sleep deprivation in Wistar rats has been linked to oxidative stress in the submandibular salivary glands.^3,4^ Similarly, a recent study has reported submandibular salivary gland tissue degeneration due to inflammatory response following a 96-hour total sleep deprivation in male Wistar rats.^5^

The natural response of the body to infection and injury is inflammation, which also serves as a critical survival mechanism in higher animals. A group of cell-derived cytokines strengthen inflammatory responses, whether acute or chronic, the activities of which determine the process and severity of inflammation. Generally, inflammations (especially in chronic conditions) are associated with inflammatory mediators and the activation of harmful signaling pathways, all of which contribute to disease progression. Thus, inflammatory biomarkers, including cellular factors such as neutrophils and lymphocytes and molecular factors such as cytokines, either in circulation or confined to tissues could indicate disease progression and severity. Studies have reported associations between sleep deprivation and certain cytokines along with other inflammatory markers.^6,7^ Indeed, studies have indicated that certain cytokines, especially interleukin-6 (IL-6), interleukin-1 (IL-1), c-reactive protein (CRP), and tumour necrosis factor-alpha (TNF-α) are important in the pathophysiology of sleep deprivation-induced pathologies.^8,9^ For example, increased levels of certain proinflammatory markers (CRP, IL-1, IL-6, and TNF-α) have been reported in individuals that experience chronic sleep deprivation.^7–9^ Both highly sensitive CRP levels and leukocyte counts were significantly higher in permanent night shift employees than in daytime workers.^10^ Similarly, higher level of serum IL-6 among night workers, when compared with regular workers, has been reported.^7^ Moreover, a recent study has reported that chronic sleep deprivation equal to eight hours a day for 20 days elevated IL-1β and TNF-α serum levels.^11^

Although a recent study has reported submandibular salivary gland tissue degeneration due to inflammatory response and cellular death following a 96-hour total sleep deprivation in rats,^5^ our previous study indicated that total sleep deprivation and partial sleep deprivation produced differing effects on salivary secretion.^3^ However, whether salivary inflammatory responses occur to the same extent in paradoxical sleep deprivation with or without sleep recovery remains unknown. Therefore, this study aimed to investigate the extent to which inflammation influences salivary impairments associated with paradoxical sleep deprivation with or without sleep recovery in male Wistar rats. Its null hypothesis suggests that partial and total sleep deprivation cause no difference in salivary inflammatory responses.

## Methodology

### Experimental design

**Figure 1:**
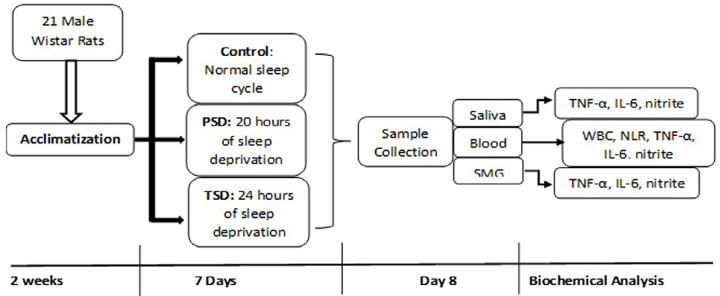
Study protocol, n=7 per group.

The study protocol followed is indicated in Figure 1. Ethical approval was issued for this study by the University of Ibadan Animal Care and Use Research Ethics Committee (UI-ACUREC/105-1121/15). Animals were purchased from the animal house of the institution. The regulations for animal use were strictly followed in accordance with the directives of the United Kingdom Animals Use Act 1986. In total, 21 male Wistar rats weighing 200-250 grams were used. Animals were allowed to acclimatize for two weeks at a standard room temperature (23-25°C), humidity (35-55%), and a natural photoperiod of 12 hours of light/dark cycle, with free access to drinking water and a standard rat pellet (Ladokun Feed^®^). Sample size was determined as previously described.^3^ Calculation of sample size followed the "resource equation method," in which, a value "E" is measured as the degree of freedom of analysis of variance (ANOVA), which should range from 10 to 20, in which E = Total number of animals – Total number of groups. The animals were randomly assigned (by simple randomization) to three groups of seven rats each as control, partial sleep deprivation (PSD), and total sleep deprivation (TSD). All animals were firstly divided into three groups according to their body weights in grams (group 1 = 200 – 219, group 2 = 220 – 239, group 3 = 240 – 250) and then simultaneously randomized into the experimental groups (Control, PSD, and TSD). The TSD group was deprived of paradoxical sleep for 24 hours, whereas the PSD group was deprived of paradoxical sleep for 20 hours and then allowed four hours of daily sleep (sleep recovery) in a plastic cage from 10:00 a.m. to 2:00 p.m. for seven days. The control animals received no sleep deprivation at any point during this study. Thus, they were allowed the normal sleep duration of an average period of 12 hours per day. They were also placed in a similar chamber without water so as to have a similar enviroment as the other groups.^3,4^

### Sleep deprivation model

The sleep deprivation model was a modified form of the model as previously described.^3^ A plastic tank measuring 110 × 90 × 35 cm with 31 metal platforms measuring 6 cm in diameter and a 25-cm high pedestal surrounded by water to about 5 cm below the platform surface was used.

### Saliva and blood collection

Following seven days of the experiment, the rats were anesthetized using ketamine (100 mg/kg, i.p.) and xylazine (5 mg/kg, i.m.) and secured on a board for saliva collection, as previously described.^12^ Stimulated saliva samples (using pilocarpine hydrochloride, 10 mg/kg, i.p.) (Alfa Aesar Fischer Scientific, UK) were taken from 8 to 10 am for 10 minutes and immediately stored at −80 °C prior to biochemical assay. Blood samples were obtained via cardiac puncture into plain sample bottles for serum biochemical assay and EDTA bottles to determine leukocyte counts.

### Submandibular salivary gland tissue collection

The submandibular glands were surgically removed and homogenized in phosphate-buffered saline (PBS, pH 7.4) as previously described to obtain supernatant which was stored at −80 °C for the biochemical assays.^4^

### Estimation of IL-6 concentration in saliva, serum, and submandibular salivary gland

The ELISA kits to estimate IL-6 concentrations were acquired from BioLegend, San Diego, CA 92121 U.S.A. All procedures were carried out in accordance with the manufacturer's guidelines. The lyophilized Mouse IL-6 Standard was reconstituted by adding 0.2 mL of 1X Assay Diluent A to make the 156.5 ng/mL standard stock solution. Moreover, 1000 μL of the top standard at 500 pg/mL was prepared by adding 3.2 μL of the reconstituted standard stock solution to 996.8 μL 1X Assay Diluent A. Then, two-fold serial dilutions of the 500 pg/mL top standard were performed with 1X Assay Diluent A in separate tubes six times. 1X Assay Diluent A served as the zero standard (0 pg/mL). Finally, 100 μL of the diluted Capture Antibody solution were added to each well and other protocols were followed as previously indicated.^4^ Absorbance was read from 450 to 570 nm.

### Estimation of TNF-α concentration in saliva, serum, and submandibular salivary gland

The ELISA kits to estimate TNF-α concentrations were acquired from BioLegend, San Diego, CA 92121 U.S.A. All procedures were carried out in accordance with the manufacturer's guidelines. Briefly, the Diluted Capture Antibody solution was added to each well, after which the plate was sealed and incubated overnight at 6° Celsius. The plates were washed and blocked by adding 200 uL 1X Assay Diluent A into each well, sealed and shaken in a 0.3-cm circular orbit for one hour at room temperature (25 °C). The plates were washed four times and filled with 100-uL diluted standards and samples in the appropriate wells. The plates were sealed and incubated for another two hours at room temperature with shaking at intervals. The plates were washed four times, and each well was filled with 100 uL of diluted Detection Antibody solution, sealed, and incubated at room temperature for one hour with shaking at intervals. Each well was filled with 100 uL of diluted Avidin-HRP solution and sealed. The sealed plates were incubated for 30 minutes at room temperature with intermittent shaking. The final washing was carried out five times, each time soaking the dishes from 30 to 60 seconds. Each well received 100 uL of freshly mixed TMB Substrate Solution and the plates were incubated in the dark for 15 minutes. Finally, 100 uL of Stop Solution (HCl) was added to each well. Absorbance was read from 450 to 570 nm.

### Estimation of nitrite concentration in saliva, serum, and submandibular salivary gland

The enzymatic conversion of nitrate to nitrite by nitrate reductase was used to detect nitric oxide concentrations. Colorimetric detection of nitrite as an azo dye product of the Griess Reaction was employed. The reagents, working standards, and samples were all prepared according to the manufacturer's guidelines (Sigma^®^, USA). Briefly, 50 uL of Reaction Diluent (diluted 1:10) were introduced to the blank wells and the remaining wells were filled with 50 uL of Nitrite Standard or sample. Each well then received 50 uL of Reaction Diluent (diluted 1:10), followed by 50 uL of Griess Reagent I to the samples and 50 uL of Griess Reagent II to each well, which were gently mixed. The samples were incubated at room temperature for 10 minutes. A microplate reader set to 540 nm was used to determine the absorbance of each well. To apply the obtained nitrite level as an index of nitric oxide concentration, the obtained values were normalized by the total protein of each sample. Total protein was determined using commercially available kit (Fortress Diagnostic Limited, Antrim, BT41 1QS, United Kingdom) according to the manufacturer's description.

### Determination of leukocyte count and neutrophil-lymphocyte ratio

Leukocyte and differential counts were determined using an automated blood cell analyzer (Sysmex America Inc., Lincolnshire, IL60069, USA).

### Data analysis

Levels of IL-6, TNF-α, nitrite, leukocyte count, neutrophil-lymphocyte ratio, and histological changes in serum, saliva, and submandibular glands were chosen as the outcome variables. Quantitative data are shown as mean ± SD (standard deviation). Data were analyzed by GraphPad Prism, version 8.0.2, for Windows 10 (GraphPad^®^ Software, San Diego, CA, USA). The Shapiro-Wilk test was used to test the assumption of normality. One-way ANOVA and Tukey's post hoc tests were used for analyses. Results with p-values 0.05 were considered statistically significant.

## Results

### Effect of paradoxical sleep deprivation on serum, saliva, and submandibular IL-6, TNF-α, and nitrite levels

**Figure 2:**
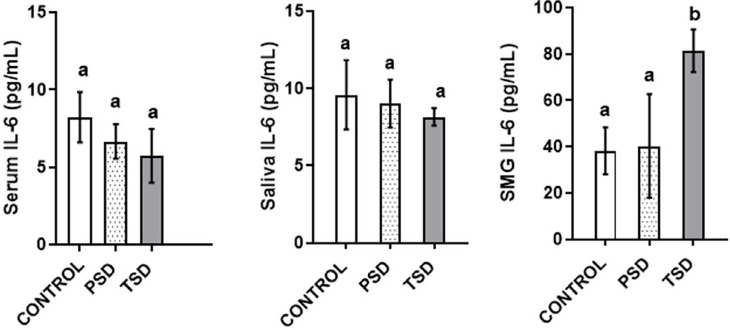
Effect of paradoxical sleep deprivation on serum (A), saliva (B), and submandibular (C) IL-6 levels, n=7 per group, values with different alphabets are significantly different. Data are shown as mean ± SD (error bar) and analyzed using the ANOVA and Tukey's post hoc tests.

The submandibular salivary glands of the animals in TSD showed significantly higher IL-6 levels (81.39±10.6 pg/mL) than those in control (38.24±10.1 pg/mL) (p<0.001). However, this study observed no significant difference in the levels of serum and saliva IL-6 between groups (p=0.52 and p= 0.82 respectively) (Figure 2).

**Figure 3:**
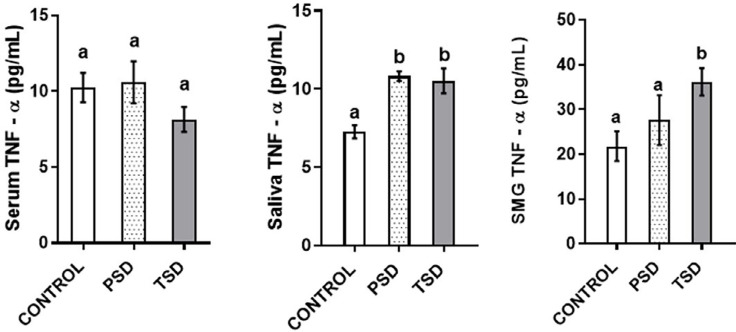
Effect of paradoxical sleep deprivation on serum (A), saliva (B), and submandibular (C) TNF-α levels, n=7 per group, values with different alphabets are significantly different. Data are shown as mean ± SD (error bar) and analyzed using the ANOVA and Tukey's post hoc tests.

The submandibular salivary glands of the animals in TSD showed significantly higher TNF-α levels (36.18±3.63 pg/mL) than those in control (21.81±3.37 pg/mL) (p=0.04). Also, PSD (10.83±0.77 pg/mL) and TSD (10.51±2.1 pg/mL) showed significantly higher TNF-α levels than the control (7.26±1.14 pg/mL) (p=0.003 and p=0.01 respectively). However, this study observed no significant difference in the level of serum TNF-α between groups (p=0.74) (Figure 3).

**Figure 4:**
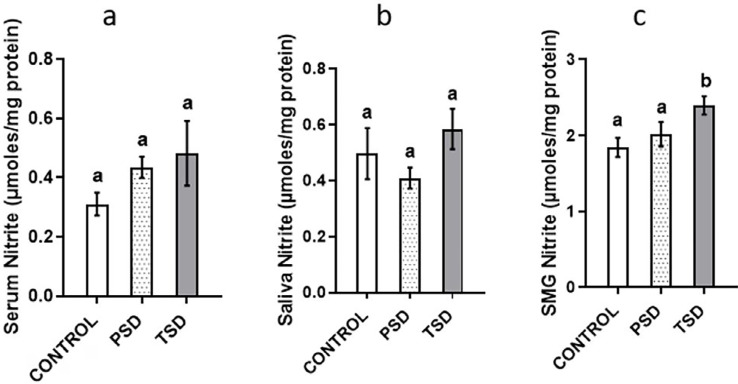
Effect of paradoxical sleep deprivation on serum (A), saliva (B), and submandibular (C) nitrite levels, n=7 per group, values with different alphabets are significantly different. Data are shown as mean ± SD (error bar) and analyzed using the ANOVA and Tukey's post hoc tests.

The submandibular salivary glands of the animals in TSD showed significantly higher nitrite levels (2.4±0.32 μmole/mg protein) than those control (1.84±0.32 μmole/mg protein) (p=0.03). However, this study observed no significant difference in the levels of serum and saliva nitrite between groups (p=0.48 and p=0.21 respectively) (Figure 4).

### Effect of paradoxical sleep deprivation on leukocyte counts and neutrophil-lymphocyte ratio

**Figure 5:**
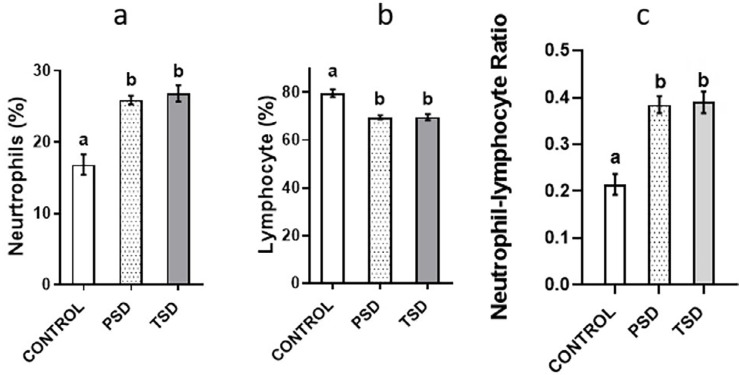
Effect of paradoxical sleep deprivation on neutrophil percent (A), lymphocyte percent (B), and neutrophil/lymphocyte ratio (C), n=7 per group, values with different alphabets are significantly different. Data are shown as mean ± SD (error bar) and analyzed using the ANOVA and Tukey's post hoc tests.

The PSD group showed a significantly higher leukocyte count (6830 X 10^3^ul ± 1242.53) than the control (4535.71 X 10^3^ul±640.98) (p=0.0004). The PSD 25.88±1.64) and TSD (26.82±3.05) showed a higher neutrophil count (percent) than the control (16.86±3.82), but a lower lymphocyte count (percent) — PSD (69.5±2.28) and TSD (69.55±3.31) — than the control (79.57±.29). Similarly, PSD and TSD showed a significantly higher neutrophil-lymphocyte ratio than the control (Figure 5).

### Effect of sleep deprivation on the submandibular gland histology

**Figure 6:**
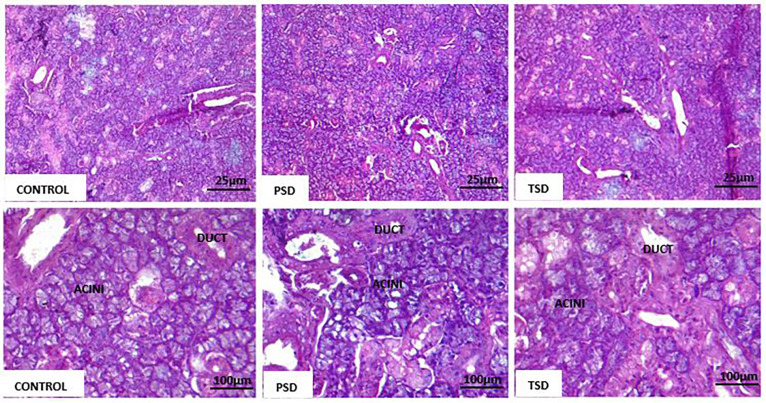
Submandibular salivary glands in Control (×100; ×400), PSD (×100; ×400), and TSD (×100; ×400) showing normal acini and ducts.

The submandibular glands from the three groups showed normal salivary gland architecture, with well-defined lobules composed of round to oval mucous and serous acinar cells interspersed with well-arranged ducts and normal fibro-vascular stroma. Inflammatory cells showed no infiltration (Figure 6).

## Discussion

This study examined the influence of inflammation on the changes in salivary secretion associated with paradoxical sleep deprivation and, more importantly, determined the extent of the involvement sleep deprivation with or without sleep recovery. Total seven-day sleep deprivation raised the levels of inflammatory cytokines (IL-6 and TNF-α) and nitrite in submandibular salivary glands. This finding supports Souza, et al.^5^ (2021), which indicated raised levels of TNF-α and IL-6 gene expression in submandibular glands after 96 hours of sleep deprivation in rats. Both TNF-α and IL-6 have been implicated in inflammatory salivary gland diseases such as Sjogren's disease and irradiation-induced sialadenitis.

While IL-6 is essential for an optimal acute-phase response after tissue damage, optimal immune response to pathogens also require it.^13^ More importantly, it is essential for mucosal immunoglobulin A production, a major antimicrobial factor in saliva.^14^ The apoptosis-suppressing effect of IL-6 is associated with the upregulation of Bcl-xL and Mcl-1 in the salivary glands.^15^ Since IL-6 has pro-inflammatory and anti-inflammatory properties, it can elicit either tissue protection or destruction. Thus, it is important to note that the overexpression of IL-6 in the submandibular salivary gland might act as a disease promoting factor or as a defense mechanism, protecting gland tissue integrity. In addition to Jak1/2-STAT3 pathway, IL-6 can also activate the Ras-Raf-MEK-Erk and PI3 kinase-Akt signaling pathways which have been shown to contribute to the anti-apoptotic function of IL-6 in different cells types.^16,17^ Hence, the role of IL-6 in a particular immunological process depends on specific disease settings, cytokine environments, and tissue- and cell-specific factors. In the salivary gland inflammation associated with Sjogren syndrome, IL-6 is crucial for suppressing apoptosis and protecting tissue integrity, whereas largely unessential for immune responses.^18^ Therefore, the overall function of IL-6 during the immunological phase of exocrine gland inflammation is both tissue-protective and anti-inflammatory depending on the stage or duration of the condition.

Generally, TNF-α enhances the secretion of acinar cell-synthesized matrix metalloproteinase-2 (MMP-2) expression. Thus, it may destroy the basement membrane and degenerate acinar cell morphology by MMP-2 modulation.^19^ Consequently, acinar cell death may release salivary gland-specific autoantigens that elicit further immune responses. Similarly, TNF-α has been shown to induce caspase-3 activation and apoptosis in salivary gland epithelial cells and disrupt the function of tight junctions, all of which will impair secretory function.^20^ TNF-α, alone or in combination with other cytokines, can induce the apoptosis of exocrine gland cells by both activating mitochondria/caspase-9-dependent pathway and inducing Fas expression to enhance Fas/Fas ligand-caspase-8-dependent cell death. Both cytokine-induced and Fas/Fas ligand-mediated apoptosis are implicated in the pathogenesis of inflammatory exocrine gland diseases (e.g., Sjogren syndrome) and are also likely involved in anti-CD3-induced apoptosis of exocrine gland tissues since anti-CD3 treatment can induce TNF-α production and Fas ligand expression by T cells.^21^

Increase in nitrite has been shown to influence inflammatory and immunological activities in the body systems.^22^ Thus, elevated nitrite in the submandibular glands of sleep-deprived rats in this investigation can explain the increased tissue inflammatory activities. Elevated concentrations of nitrite, a nitric oxide metabolite, has been shown for various inflammatory conditions.^23,24^ Nitric oxide in the inflamed tissue controls the synthesis of several inflammatory mediators and functions of inflammatory cells. The role of nitrite/nitric oxide in inflammation is either protective or destructive depending on the type and phase of the inflammatory process.^25^ For example, nitric oxide mediates some of the damaging effects in proinflammatory cytokines such as interleukins.^25^ The conversion of nitric oxide to nitrite can occur by several pathways such as the xanthine oxidoreductase, aldehyde oxidase, and cytochromes pathways.^26^ Importantly, nitrite without reduction to nitric oxide can stimulate intracellular processes such as activation of adenylate kinase-AMPK signaling and modulating nuclear factor-kappa B and soluble guanylate cyclase.^27^ Also, nitrite, independent of nitric oxide, has been shown to directly nitrosylate a variety of pathways, including heme oxygenase-1, heat shock protein 70, and cytochrome P450 activity.^28^ Furthermore, nitrosylation and generation of nitric oxide species have been shown to injure tissue. For example, the presence of oxidants such as superoxide radicals and hydrogen peroxide can oxidize nitric oxide to peroxynitrite anion and nitrogen dioxide.^29^ This can generate nitrotyrosine, which has been implicated in various diseases. Hence, nitrite and nitric oxide have a myriad of potential targets by a variety of mechanisms that can be explored to better understand different disease conditions, including salivary gland disorders.

Reports on circulating levels of IL-6 and TNF-α after sleep deprivation are generally inconsistent.^30,31^ This study observed no difference in IL-6, TNF-α, and nitrite serum levels but their higher levels in the submandibular glands of the evaluated rats may stem from the local production of these cytokines. Studies have reported that salivary gland acinar cells synthesize and store interleukins as cytoplasmic granules which are released after stimulation.^32^ Submandibular salivary gland cells expressed similar levels of IL-6Rα as splenocytes, which are mostly IL-6Rα-expressing lymphocytes, suggesting that submandibular salivary gland cells express abundant endogenous IL-6Rα to receive IL-6 signaling in a conventional fashion.^18^ Likewise, IL-6 treatment induced the activation of STAT3 in *in vitro* human submandibular salivary gland cells, further supporting that IL-6 can directly act on salivary gland epithelial cells.^18^

The type of used saliva may explain regarding the lack of changes in the saliva levels of IL-6 and nitrite against their elevated levels in submandibular glands following total sleep deprivation. All salivary glands, including major and minor ones, contribute to saliva, which might have altered cytokine levels in saliva. Thus, a gland-specific saliva sample could have produced a different result. A previous study in humans has also reported no difference in salivary IL-6 and TNF- levels between day and night shift workers.^33^

Regarding the other inflammation markers in this study, the sleep deprived groups showed higher neutrophil counts and neutrophil-lymphocyte ratios, in agreement with previous studies in human and animals.^34,35^ Neutrophils have been established to play a significant role in acute and chronic inflammation. Inflammatory processes mediate the increase in neutrophil count by producing and releasing various cytokines, of which many have been implicated in sleep deprivation.^36^ Moreover, the sleep deprived groups showed decreased circulating lymphocyte levels, which agrees with previous findings.^37^ However, the neutrophil-lymphocyte ratio has been shown to configure a good prognostic value for the risk of inflammatory diseases, and elevated levels of it have been associated with these conditions.^38^

Importantly, the histological analysis of the submandibular salivary glands in this study showed no morphological abnormalities between the groups. Specifically, the sleep deprivation group showed no inflammatory cell infiltration. This study used seven days of paradoxical sleep deprivation, which may inadequately assess signs of chronic inflammatory response by the presence of chronic inflammatory cells and other features, such as acinar degeneration and periductal fibrosis. This finding seems appropriate because elevated levels of cytokines in the salivary glands have been reported to occur before (about one week) infiltration by chronic inflammatory cells that occurs later (about eight weeks).^39^ For example, the elevated levels of TNF-α and IL-6 in the parotid gland acinar cells of aging mice preceded the periductal lymphoid aggregates and acinar cell secretory dysfunction.^40^

This study has some limitations, including its lack of assessment of all major salivary glands. Although submandibular gland is a major gland that significantly contributes to salivary secretion. The evaluation of other salivary glands might change our observations. This study also assessed only few markers of inflammation and oxidative stress. Analysis of more markers and other molecular mechanisms underlyng the inflammatory response could provide further insight to our findings. Moreover, while rat salivary glands have been shown to both anatomically and functionally have similarities with human salivary glands, our findings may be inapplicable to humans directly. Some genetic and environmental factors such as diet and lifestyles may affect findings in humans, which may prevent extrapolation to human, hence the need for further studies on these issues.

In this study, partial seven-day sleep deprivation failed to upregulate the inflammatory process markers in submandibular salivary glands, unlike total sleep deprivation. In the same vein, we reject our null hypothesis of no difference between the extent to which partial and total sleep deprivation affect salivary inflammatory response. This finding corroborates reports on the ameliorative effect of sleep recovery on various pathologic conditions, including the reversal of impaired salivary secretion previously reported in our study.^3^

## Conclusion

Total sleep deprivation for seven days was associated with higher levels of inflammatory markers in submandibular salivary glands, whereas partial sleep deprivation for the same duration showed no similar effect. This may indicate that four-hour sleep recovery prevented inflammatory responses in submandibular salivary glands.

## Data Availability

All data generated or analyzed in this study are included in this published article
